# Acute effects of commercial energy drink consumption on exercise performance and cardiovascular safety: a randomized, double-blind, placebo-controlled, crossover trial

**DOI:** 10.1080/15502783.2023.2297988

**Published:** 2024-01-10

**Authors:** Nile F. Banks, Emily M. Rogers, Nate J. Helwig, Laura E. Schwager, Justin P. Alpers, Sydni L. Schulte, Emma R. Trachta, Christopher M. Lockwood, Nathaniel D.M. Jenkins

**Affiliations:** a University of Iowa, Integrative Laboratory of Applied Physiology and Lifestyle Medicine, Iowa City, IA, USA; b Dr. Chris Lockwood, LLC, Casper, WY, USA; c University of Iowa, Abboud Cardiovascular Research Center, Iowa City, IA, USA

**Keywords:** Caffeine, maximal oxygen uptake, muscular endurance, blood pressure, ventricular repolarization

## Abstract

The aim of this study was to examine the acute effects of a non-caloric energy drink (C4E) compared to a traditional sugar-containing energy drink (MED) and non-caloric placebo (PLA) on exercise performance and cardiovascular safety. Thirty healthy, physically active males (25 ± 4 y) completed three experimental visits under semi-fasted conditions (5–10 h) and in randomized order, during which they consumed C4E, MED, or PLA matched for volume, appearance, taste, and mouthfeel. One hour after drink consumption, participants completed a maximal, graded exercise test (GXT) with measurement of pulmonary gases, an isometric leg extension fatigue test (ISO_FTG_), and had their cardiac electrical activity (ECG), leg blood flow (LBF), and blood pressure (BP) measured throughout the visit. Neither MED nor C4E had an ergogenic effect on maximal oxygen consumption, time to exhaustion, or peak power during the GXT (*p* > 0.05). Compared to PLA, MED reduced fat oxidation (respiratory exchange ratio (RER) +0.030 ± 0.01; *p* = 0.026) during the GXT and did not influence ISO_FTG_ performance. Compared to PLA, C4E did not alter RER (*p* = 0.94) and improved impulse during the ISO_FTG_ (+0.658 ± 0.25 V·s; *p* = 0.032). Relative to MED, C4E did not significantly improve gas exchange threshold (*p* = 0.05–0.07). Both MED and C4E increased systolic BP at rest (+7.1 ± 1.2 mmHg; *p* < 0.001 and + 5.7 ± 1.0 mmHg; *p* < 0.001, respectively), C4E increased SBP post-GXT (+13.3 ± 3.8 mmHg; *p* < 0.001), and MED increased SBP during recovery (+3.2 ± 1.1 mmHg; *p* < 0.001). Neither MED nor C4E influenced ECG measures (*p* ≥ 0.08) or LBF (*p* = 0.37) compared to PLA. C4E may be more efficacious for improving performance in resistance-type tasks without altering fat oxidation under semi-fasted conditions during fatiguing exercise bouts, but promotes similar changes in BP and HR to MED.

## 1. Introduction

Caffeine-based energy drink consumption has increased dramatically over the last two decades, partially driven by new marketing strategies highlighting their claimed ergogenicity related to exercise performance [1]. Energy drinks are often marketed toward young adults who may be most interested in these benefits [2]. Many commercially available energy drinks contain a blend of ingredients that include taurine, glucuronolactone, and B vitamins, with many containing ≥3.3 g of added sugar per fluid ounce. However, sugar-free energy drink formulations that contain negligible kilocalories and include sport supplements such as beta-alanine and L-citrulline are becoming increasingly popular, particularly due to their purported physical performance benefits.

It has been reported that traditional caffeine-based energy drinks improve time trial performance [3,4], submaximal aerobic performance [5], and potentially muscular strength and endurance [6–8], but not maximal exhaustive exercise [9]. The limited data on the effects of non-caloric energy drink consumption on exercise performance suggest that these drinks may be less effective than high-sugar containing energy drinks [10–12]. Alternatively, Souza *et al*. [13] reported that improvements in exercise performance following acute consumption of sugar-containing energy drinks is associated with the product’s taurine dosage. Further, it is not clear how the addition of ingredients such as beta-alanine and L-citrulline to novel non-caloric energy drink formulations influences efficacy. However, there is reason to hypothesize that these formulations may provide performance benefits more similar to high-sugar containing energy drinks. For example, although the greatest performance benefits of ingredients such as beta-alanine and L-citrulline require habitual supplementation due to their primary mechanism(s) of action (e.g. augmenting carnosine [14] and endothelial nitric oxide bioavailability [15,16]), there is also some evidence that acute dosing of these ingredients may promote exercise performance. For example, acute beta-alanine consumption has been shown to reduce perceived exertion during repeated Wingate cycling tests [17], and the limit time at maximum aerobic velocity [18] and the distance run during 6-min run tests in endurance athletes [19]. Together, these data suggest that the efficacy of energy drinks cannot be ascertained based on their caffeine-content (or any single ingredient) alone, but instead should be studied as finished products in order to accurately gauge efficacy as used by the consumer.

Given the rise in caffeine-based energy drink consumption, there is also a great need to understand their impact on cardiovascular health. Multiple studies have now indicated that consumption of traditional caffeine-based energy drinks may cause prolongation of the QTc interval [20,21], which primarily measures ventricular repolarization. Prolongation of the QTc interval is an important safety concern because small perturbations can trigger malignant arrhythmias [22]. Further, traditional caffeine-based energy drink consumption has been consistently shown to cause elevations in resting systolic and diastolic blood pressure (SBP and DBP) [20,21,23,24], although it is unclear if energy drink consumption amplifies exercise-associated increases in blood pressure, heart rate (HR), or myocardial oxygen demand. Caffeine alone has also been shown to interfere with post-exercise autonomic recovery via increased sympathetic activity [25], and thus energy drink consumption may impede cardiovascular recovery following exhaustive exercise. Finally, while the caffeine content of energy drinks is likely largely responsible for these cardiovascular effects with the most pronounced effects occurring with high volume consumption (e.g. ≥750 mL with ≥240 mg caffeine) [20,21], it appears that there is a synergistic effect of common energy drink ingredients on parameters such as the QTc interval, SBP, and DBP [21]. Thus, it is critical that studies examine the acute effects of energy drink consumption on cardiovascular safety profiles at rest and following exercise to understand the potential cardiovascular risks associated with their consumption, particularly if used to improve exercise performance.

Therefore, we compared the effects of acute consumption of commercially available sugar-free (C4 Energy™) versus traditional (Monster Energy™) energy drinks versus placebo on (1; *Primary Outcome*) maximal exercise performance during graded exercise to volitional exhaustion (2; *Primary Outcome*) the ventilatory threshold and fuel utilization during graded exercise (3; *Primary Outcome*) performance as demarcated by the total impulse (force ×time) completed during submaximal isometric exercise to volitional exhaustion, and (4; *Secondary Outcome*) cardiovascular safety parameters (e.g. blood pressure, heart rate, rate pressure product, and QTc interval length) at rest, following exercise, and in post-exercise recovery. Other secondary outcomes included leg blood flow and mood, while perceived effort during the testing sessions, blinding efficacy, and adverse events were included as tertiary outcomes.

## 2. Methods

### 2.1. Experimental design

This study employed a randomized, double-blind, placebo-controlled, crossover design where each subject completed three identical 2.5-hour experimental visits. The only difference between visits was the drink consumed, where either a non-caloric energy drink containing a novel ingredient blend including caffeine (C4E), a traditional energy drink that included caffeine and sugar (MED), or a PLA which was non-caloric and contained no caffeine or active ingredients was consumed. Each of these visits were performed at the same time of day (±90 min) and were separated by 7 ± 3 days. Before attending the first experimental visit, participants completed a familiarization visit to become acquainted with each of the tests performed.

**Figure 1.**
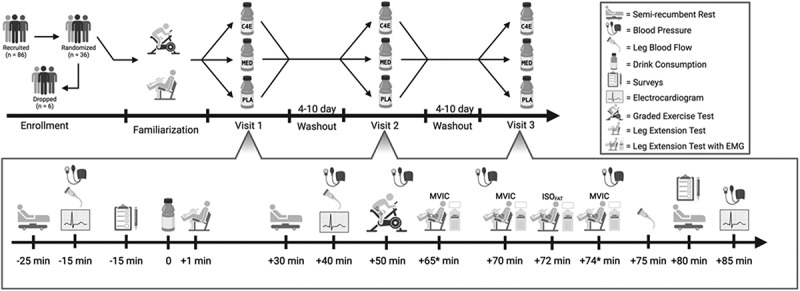
Detailed overview of the experimental design and visits. The time scale for experimental visits is depicted relative to the time of drink consumption (time = 0). *These tests were performed immediately following the preceding exercise test. Thus, timing varied slightly between participants and within visits depending on the time to exhaustion during the graded exercise test and time to fatigue during the isometric fatigue protocol. EMG = Electromyography; ISO_FTG_ = isometric fatigue protocol; MVIC = maximal voluntary isometric contraction; C4E = C4 energy drink; PLA = Placebo; MED = monster energy caffeine drink. Figure created with BioRender.com.

Prior to each experimental visit, participants abstained from exercise, caffeine, and alcohol for 24 h, and arrived at the laboratory following a 5–10 h fast. At the beginning of the experimental visits, participants provided a urine sample which was analyzed for urine-specific gravity using handheld refractometry (Fisherbrand, Pittsburgh, PA) to ensure they were euhydrated. The study visit commenced only if the participant’s urine-specific gravity was between 1.005 and 1.030. Participants then laid down on a padded examination table in a semi-recumbent position and rested quietly for 10 min. After 10 min, femoral artery blood flow (LBF) was measured, an electrocardiogram (ECG) recording was obtained, and resting blood pressure and oxygen saturation levels were measured. Prior to drink consumption, participants completed the POMS-SF to assess mood. Participants then consumed the assigned drink in under 5 min. Both the participants and the researchers collecting data were blinded to the drink being consumed. Participants then immediately performed two maximum voluntary isometric contractions (MVIC) to assess maximal voluntary force (MVF) of the knee extensors before the active ingredients in the drinks had time to be metabolized. Participants were allowed to rest for 30 min, before laying back down in a semi-recumbent position on the padded examination table and resting quietly for 10 min. At 40-min post-drink consumption, femoral artery LBF, blood pressure, and oxygen saturation were measured, and another ECG recording was obtained. Participants then performed a graded maximal exercise test (GXT) on a cycle ergometer, immediately followed by another MVIC. They were then given a 5-min rest before performing 2 more MVICs, followed by an isometric fatigue test 1 min after the second MVIC. After participants finished the isometric fatigue test, they performed one last MVIC and then were promptly moved back to the padded examination table for a final assessment of femoral artery LBF. Participants were then allowed to rest quietly for 5 min while completing post-exercise surveys, including the POMS-SF and an adverse events questionnaire. After this, their blood pressure and oxygen saturation were measured and their ECG was recorded for the final time. At the end of the final visit, participants also completed a questionnaire to assess blinding efficacy. An overview of the experimental design and each experimental visit is shown in Figure 1. This trial is registered at ClinicalTrials.gov (NCT05559372).

### 2.2. Participants

**Figure 2.**
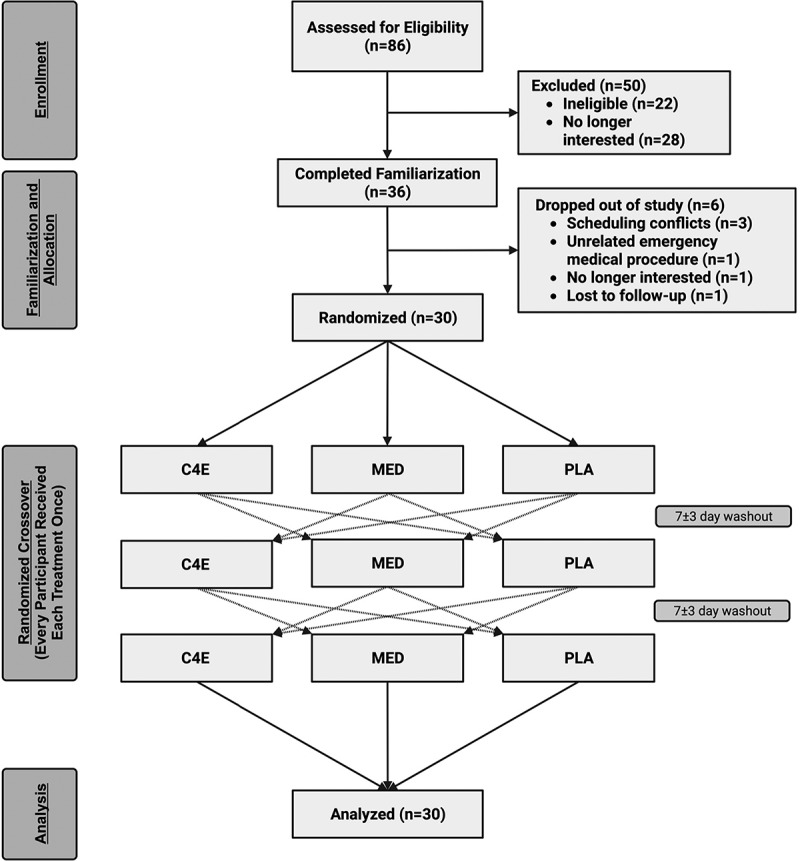
CONSORT flow diagram depicting the flow of participants through the study from initial screening to data analysis. Figure created with BioRender.com.

Thirty healthy, physically active, young adult males completed this study. An overview of participant flow from initial eligibility to data analysis is provided in Figure 2. Prior to enrollment, participants completed an informed consent form and health history questionnaire. To be eligible, participants must have been aged 20–35 years, been exercising at least 3 days per week, not been consuming more than 21 servings of caffeine per week, had a body mass index of 18.5–34.9 kg/m^2^, and been healthy according to self-reported health history. Participants were recruited through approved e-mails using the university mass-e-mail system, flyers placed across campus, and by word of mouth. All study procedures and documents complied with the Declaration of Helsinki and were approved by the University’s Institutional Review Board for the Protection of Human Subjects (IRB Approval #: 202107364). All participants consented to participation by signing an informed consent form prior to participation.

### 2.3. Drink composition and Blinding

Beverages were matched for volume (16 fl oz), appearance, aroma, taste, and texture. The high-calorie, high-sugar treatment beverage (MED; Monster Energy_®_, Monster Energy Company, Corona, CA) was purchased in case packs from a big box retailer in Iowa City, IA, placed in refrigeration, and unopened until immediately before subject consumption. A researcher or graduate student un-involved in data collection or analysis who was unblinded to the MED beverage was responsible for pouring all subject treatment beverages into drinking cups. This process occurred within a separate storage room so that other researchers and participants remained double-blinded across all randomized, crossover treatments.

The non-caloric, no sugar treatment beverage (C4E; C4 Energy™, Nutrabolt_®_, Austin, TX) and PLA were additionally double-blinded for final packaging and formulation adjusted to organoleptically match the MED beverage. MED supplied 230 kcals, 58 g Total Carbohydrate (54 g added sugars), and included the active ingredients: Sugar, Glucose [D-glucose (Dextrose) or DL-glucose form undisclosed], Taurine, Carnitine (as L-Carnitine L-Tartrate), 160 mg Caffeine, Niacin (30 mg as Niacinamide), Inositol, Vitamin B6 (3.8 mg as Pyridoxine HCl), Maltodextrin, Vitamin B12 (12 mcg as Cyanocobalamin), and Vitamin B2 (3.3 mcg). C4E and PLA contained equivalent energy, acidulants, and inactive ingredients. C4E and PLA supplied < 5 kcals, 0 g Total Carbohydrate (0 g added sugars), and C4E included the active ingredients: Beta-Alanine (CarnoSyn_®_, Natural Alternatives International, Inc., Carlsbad, CA), L-Citrulline, Betaine (as anhydrous BetaPower_®_, International Flavors & Fragrances Inc., New York, NY), 200 mg Caffeine (as anhydrous), Tyrosine (as N-Acetyl-L-Tyrosine), Niacin (30 mg as Niacinamide), and Vitamin B12 (6 mcg as Cyanocobalamin). Nutrition Facts panels and ingredients (in order of highest to lowest concentration) for C4E and MED are provided in Supplemental Table 1.

Finished products were independently confirmed by DYAD Labs (Salt Lake City, UT) for nutrition facts, physical properties, organoleptics, and specification identity, composition, potency, and purity using validated analytical methods. The absence of the World Anti-Doping Agency (WADA) banned substances was independently confirmed by LGC Science, Inc. (Informed Sport; Lexington, KY). Sample quantities for analytical laboratory testing were calculated using square root of n + 2, and all samples were randomly selected and then shipped using next day delivery to the respective analytical laboratories. DYAD Labs analytical results for MED utilized LOT # J2101G–0442, and LGC Sciences results for MED were analyzed against LOT # J2100G–0441. All analytical results were reviewed by CML prior to the beverages being approved for use in the present study. Both C4E and PLA beverages were manufactured at a US FDA cGMP-compliant facility. The third-party manufacturer, grant donor, and CML formulated and maintained blinding of groups, and each beverage was randomly assigned an item number by the manufacturer. Blinding was maintained until data collection and statistical analysis was completed. CML was not involved in data collection or statistical analysis.

### 2.4. Graded, maximal aerobic exercise test

Sixty-two ±8 min after drink consumption, participants completed an incremental, maximal, GXT on a cycle ergometer (Monark 939E; Monark Exercise AB, Vansbro, SWE) at a pedal cadence of 70 revolutions per minute (rpm). Following a 2-min warm up at 25 W, the power output increased to 50 W for 2 min, and then by 30 W every 2 min thereafter until the participant could no longer maintain ≥ 65 rpm cadence. Prior to each GXT, participants were reminded of the instructions of the test and were strongly encouraged to provide a maximal effort, but no verbal encouragement was provided during the GXT to ensure a consistent external environment between visits. During the GXT, all participants wore a nose clip and breathed through a two-way valve (2700, Hans Rudolph, Kansas City, MO). Expired pulmonary gases were collected and analyzed using a calibrated metabolic cart (TrueOne 2400, Parvo Medics, Sandy, UT). Twenty-second rolling-average filters were applied to the raw breath-by-breath, expired O_2_ and CO_2_ data, and then used to determine the highest relative (ml·kg^−1^·min^−1^) oxygen uptake ($\dot{V}$O_2peak_) and both peak and average respiratory exchange ratio (RER) achieved during the test. Time to exhaustion (GXT_TTE_) was determined as the point at which the participant could no longer maintain ≥ 90% of the assigned workload. Maximal fat oxidation (MFO; g/min) is the highest fat oxidation rate achieved during the GXT. To determine MFO, fat oxidation rate was calculated using the stoichiometric equation [26–28]$,1.695\times \dot{V}O2(L/min))-(1.701\times \dot{V}CO2(L/min)$, and then a 3^rd^ degree polynomial curve was fit to the relationship between fat oxidation rate (g/min) and percentage of maximal oxygen uptake (%$\dot{V}O2peak$) to generate a fat oxidation versus exercise intensity curve. Only data between the start of exercise at 50 W and when participants reached 80% of their $\dot{V}$O_2peak_ were used in the construction of the fat oxidation curve [29]. The peak fat oxidation rate was determined as the MFO. Determination of $\dot{V}$O_2peak_, RER, GXT_TTE_, and MFO were performed using custom-written LabVIEW software (v. 21, National Instruments Corp, Austin, TX). In addition, the gas exchange threshold (GET) was determined using the parametric global optimization method that determined the GET as the average threshold using the excess CO_2_ [30] and V-slope [31] methods to enhance optimize GET determination and subsequently the validity and reliability [32,33]. GET analyses were performed using the custom MATLAB script (R2020a, The MathWorks, Inc., Natick, MA) described by Kim *et al*. [33], which was further modified to express GET in absolute and relative time (min), $\dot{V}O2$ (L/min and ml·kg^−1^·min^−1^), HR (bpm), and power (W).

### 2.5. Electromyography

A wireless surface electromyographic (EMG) sensor (Delsys Trigno; Delsys Inc., Nantic, MA) was placed on the vastus laterals (VL) to quantify EMG amplitude during the isometric fatigue test (ISO_FTG_). EMG sensors were placed as we have previously described [34,35]. All EMG signals were sampled simultaneously at 2 kHz using PowerLab data acquisition hardware (AD Instruments, Colorado Springs, CO). Sampled signals were then recorded to a desktop computer and processed offline using custom-written LabVIEW software (National Instruments, Austin, TX). EMG signals were amplified using the built-in sensor amplifier with a gain of 10 VN ±1%, a common-mode rejection ratio of −80 DB, and an input impedance of > 101 5Ω//0.2 pF. EMG signals were then zero-meaned and bandpass filtered offline (10–499 Hz) using a zero-phase shift, 4^th^-order Butterworth filter before analyses.

### 2.6. Isometric fatigue test

Participants completed an isometric, constant force fatigue test at 40% of the highest MVF recorded during the two MVIC attempts completed 5 min after completion of the GXT. A force trajectory and real-time force feedback was provided on an external computer monitor placed in front of the participant throughout the test. Time to fatigue (TTF_ISO_) was determined objectively as the difference in time between initially achieving 95% of the target force and when force output fell below 95% of target torque. Impulse was calculated as the product of force and time during the maintenance of target force (Vs). EMG amplitudes (EMG_AMP_) were calculated as the root mean square (RMS) amplitudes (mV) during 10% epochs (e.g. 0–10%, 10–20%, 20–30%, etc.) of the TTF_ISO_, and normalized to the maximal EMG_AMP_ obtained during the preceding MVIC attempts.

### 2.7. Leg blood flow

All LBF measurements were obtained while participants laid supine in a semi-recumbent position with the trunk position at a ~ 35° angle in a temperature-controlled (20–22°C) quiet room. The first two LBF measurements were obtained after 10 min of quiet rest, whereas the post-exercise LBF measurement was obtained as quickly as possible following exercise cessation. A high-resolution duplex ultrasound (Logiq P9 R2.5, GE Healthcare, MA) and 12-MHz linear array transducer were used to visualize the superficial femoral artery and measure blood velocity, and a 3-min video was recorded. These videos were analyzed offline using semi-automated, continuous wall-tracking software (FMD Studio, Quipu, Pisa, Italy). Blood velocity was measured by selecting a region of the Doppler waveform and the trace of the velocity-time integral was then used to calculate mean blood velocity [36]. The following formula was then used to calculate mean femoral artery blood flow at each of the 3 measurement timepoints per visit:

$$\left(Mean\;Blood\;Velocity\;(cm/s)\times artery\;cross\;sectional\;area\left(cm^{2}\right)\right)\times 60$$

### 2.8. Electrocardiogram

A 12-lead ECG (CardioTech SE-12 Series, Edan Instruments, Pingshan District, P.R. China) was used to examine cardiac electrical activity. Following stabilization of the ECG signal, a continuous 10-s ECG recording was collected and used for analysis. Corrected QT interval (QTc, ms) was the main outcome of interest from the ECG recordings. PR interval (ms), QRS interval (ms), and P-R-T-axes (degrees) were all secondary outcomes derived from the ECG recordings.

### 2.9. Blood pressure and heart rate

Blood pressure was recorded using an automated blood pressure device (OMRON Model BP5450, OMRON Healthcare Co., Kyoto JPN) in either a semi-recumbent position, or while seated on the bike and isometric dynamometer during the GXT and ISO_FTG_ protocols Figure 1. Wherever possible, blood pressure was collected in accordance with the American Heart Association guidelines [37]. During each blood pressure recording, SBP, DBP, and HR were recorded, and rate pressure product (RPP) was derived from the product of SBP and HR at each timepoint.

### 2.10. Mood and session RPE

Mood was assessed using the POMS-SF, a 35-item 5-point Likert scale survey which assesses Total Mood Disturbance and the following six sub-scales: Fatigue-Inertia, Vigor-Activity, Tension-Anxiety, Depression-Dejection, Anger-Hostility, and Confusion-Bewilderment [38]. Session RPE was used to assess the overall intensity of effort during each visit. Specifically, following each visit, participants were asked to report how hard they felt each visit was on a 0–10 scale, with 0 representing rest and 10 representing maximal effort [39].

### 2.11. Lifestyle controls

During the 7 ± 3 day period before each experimental visit, participants were asked to fill out the Sleep Foundation Sleep Log [40] to assess sleep quantity (h/day), quality (1–5 scale), and fragmentation (awakenings/night). The Centers for Disease Control Physical Activity Diary [41] was used to assess physical activity levels and reported as minutes of daily physical activity in metabolic equivalents of task (MET·min/day). A 3-day dietary log was used to assess dietary intake, and total daily average energy consumption (kcals), as well as carbohydrate (g), fat (g), and protein (g) intake were quantified using ESHA’s Food Processor® nutrition analysis Software (https://www.esha.com, ESHA Research, Oak Brook, Il, USA), across the 3 days before each experimental visit. Subjects were provided with a copy of their 24-h pre-testing nutrition log and asked to repeat their 24-h pre-testing dietary intake prior to each subsequent crossover visit.

### 2.12. Adverse events

Immediately following each experimental visit, participants completed an adverse events survey regarding their experience following test beverage consumption for that particular visit. Participants selected either “yes” or “no” to questions asking if they experienced each of the following: nausea, vomiting, headache, stomachache/bloating/gas, diarrhea, constipation, itching, fatigue, heart palpations, or other. If “yes” was selected, participants were then asked to mark how likely it was that the adverse event/symptom was caused by the treatment beverage from the following selections: possible, likely, or very likely.

### 2.13. Blinding efficacy

Immediately after completion of the final visit, participants were told that the 3 beverages they consumed were MED, C4E, and PLA, and were asked to indicate in which visit they believed they had consumed each test beverage to the best of their ability.

### 2.14. Statistical analysis

Data that violated sphericity were corrected using the Greenhouse–Geisser correction. The effects of the MED, C4E, and PLA on $\dot{V}$O_2peak,_ GXT_TTF_, peak HR, GXT peak power, GET, MFO, average RER, mean force, TTF_ISO,_ and impulse were analyzed using independent, one-way repeated measures ANOVAs or, where data points were missing, using repeated measures mixed-effects models. Repeated measures mixed-effects models were used to analyze changes in EMG_AMP_ during the ISO_FTG_ protocol (condition (MED vs. C4E vs. PLA) x time (0–10%, 10–20%, 20–30%, … 90–100%)) and for MVIC data (condition (MED vs. C4E vs. PLA) x time (Pre-Drink, Post-GXT, Pre-ISO_FTG_, Post-ISO_FTG_)). Follow-up analyses for the EMG_AMP_ versus normalized time relationships during ISO_FTG_ included linear regression analyses with comparisons of the slopes and intercepts among conditions (MED vs. C4E vs. PLA). One-way ANOVAs or, where any data points were missing, repeated measures mixed-effects models were also used to examine if there were differences in sleep, physical activity, or diet leading up to each condition. Data are reported as mean difference ± SE of difference unless denoted otherwise and the significance was set at *p* ≤ 0.05. Statistical analyses were performed and figures were created using GraphPad Prism for macOS (v. 8.4.3).

## 3. Results

Baseline participant characteristics are presented in Table 1. Due to technical issues, data were missing at random from 4 ISO_FTG_ tests, 1 MVIC test, and 3 blood pressure tests were not available for analyses, in which case repeated-measures mixed-effects models were used.

**Table 1.** Participant characteristics (*n* = 30).

Characteristic	Mean ± SD
Age (y)	24.8 ± 4.4
Height (m)	1.8 ± 0.1
Weight (kg) BMI (kg/m^2^)	83.9 ± 14.7 26.0 ± 3.6
SBP* (mmHg)	123.7 ± 7.7
DBP* (mmHg)	72.3 ± 6.7

BMI = body mass index; SBP = systolic blood pressure; DBP = diastolic blood pressure; * = measurements obtained while participant was in a semi-recumbent position.

### 3.1. Lifestyle controls

There were no differences in total energy (kcals), fat, carbohydrate, or protein intake, nor in caffeine consumption during the 3 days leading up to each condition (all *p* ≥0.14). Further, no differences in physical activity, sleep duration, sleep quality, or sleep fragmentation were observed prior to any condition (all *p* ≥ 0.40). Lifestyle control data are presented in Table 2.

**Table 2.** Lifestyle control data during the days prior to completion of each condition.

	PLA	MED	C4E	*p*-value
Kcal	2156 ± 538	2126 ± 525	2232 ± 795	0.70
Fat (g)	88 ± 23	81 ± 26	78 ± 29	0.14
Protein (g)	104 ± 43	106 ± 36	107 ± 43	0.95
Carbohydrates (g)	236 ± 82	231 ± 72	258 ± 1	0.21
Caffeine (mg)	87 ± 102	91 ± 115	95 ± 104	0.88
Physical Activity (MET·min)	1788 ± 1984	1580 ± 1420	1344 ± 1006	0.38
Sleep Duration (h)	7.45 ± .84	7.27 ± 0.75	7.33 ± 0.90	0.41
Sleep Quality (a.u.)	3.69 ± .62	3.72 ± 0.63	3.92 ± 0.64	0.13
Sleep Fragmentation (awakenings/night)	0.76 ± .67	0.76 ± 0.76	0.73 ± 0.77	0.86

All data are presented here as mean ± SD. PLA = Placebo; MED = Monster Energy Caffeine Drink; C4E = C4 Energy Drink; MET = metabolic equivalent of task.

### 3.2. Blinding efficacy

Participants correctly determined that they had consumed PLA, MED, and C4E 67, 47, and 50% of the time, respectively.

### 3.3. Adverse events

Adverse events indicated by participants are reported in Table 3.

**Table 3.** Adverse events and self-assessed relationship to beverage consumption.

Adverse Event	PLA n (%) Reported	PLA Average Likelihood	MED n (%) Reported	MED Average Likelihood	C4E n (%) Reported	C4E Average Likelihood
Nausea	1 (3.3%)	1.0	1 (3.3%)	2.0	2 (6.7%)	1.5
Vomiting	0 (0%)	-	0 (0%)	-	0 (0%)	-
Headache	4 (13.3%)	1.0	2 (6.7%)	1.0	3 (10%)	1.7
SA/B/G	0 (0%)	-	2 (6.7%)	2.0	2 (6.7%)	1.0
Diarrhea	0 (0%)	-	0 (0%)	-	1 (3.3%)	3.0
Constipation	0 (0%)	-	1 (3.3%)	2.0	0 (0%)	-
Itching/Paresthesia	1 (3.3%)	3.0	3 (10%)	1.0	9 (30%)	2.0
Fatigue	8 (26.7%)	1.1	6 (20%)	1.2	7 (23.3%)	1.3
Heart Palpitations	0 (0%)	-	0 (0%)	-	0 (0%)	-
Other	1 (3.3%)	1.0	2 (6.7%)	1.5	4 (13.3%)	1.5

The total number and % of participants who reported an adverse event in each of the respective categories after each condition are displayed here as n and %, respectively. Average likelihood that the adverse event was caused by the beverage consumed was calculated as the mean of survey response options “Possible,” “Likely,” and “Very Likely,” which were scored 1, 2, and 3, respectively. SA/B/G = stomachache/bloating/gas; PLA = Placebo; MED = Monster Energy Caffeine Drink; C4E = C4 Energy Drink.

### 3.4. Gxt

**Figure 3.**
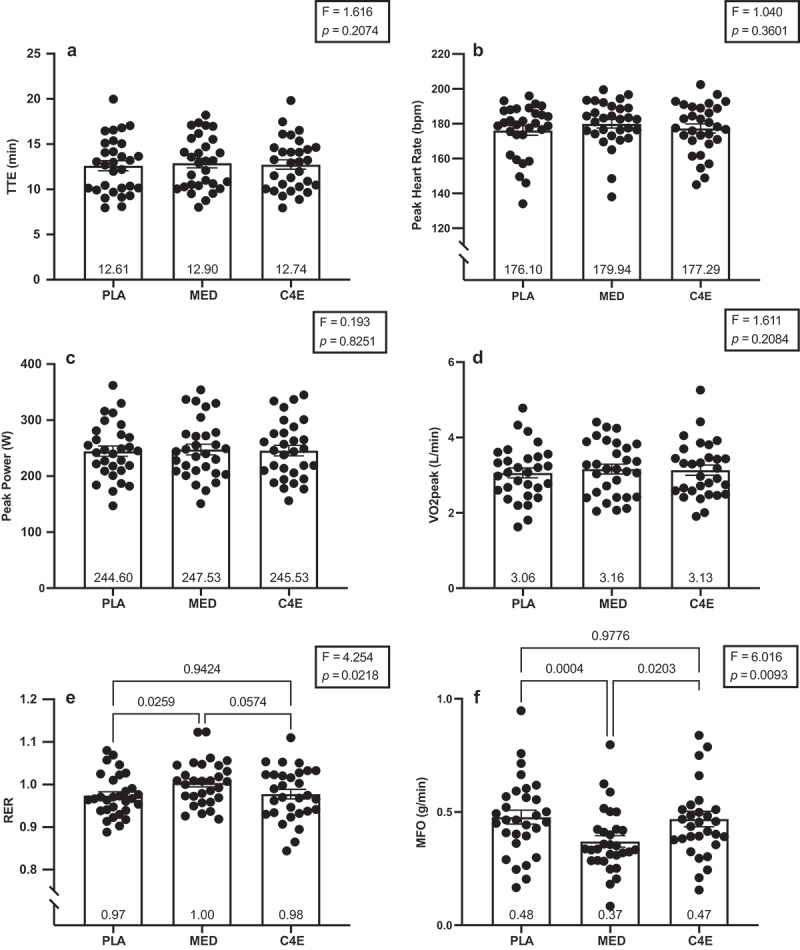
Effects of energy drink consumption maximal performance markers during graded exercise testing. The (a) time to exhaustion (TTE), (b) peak heart rate, (c) peak power, (d) $\dot{V}$O_2peak_, (e) average respiratory exchange ratio (RER), and (f) maximal fat oxidation (MFO) during the graded exercise test (GXT) following consumption of 16 oz placebo (PLA), monster energy drink (MED), and C4 energy drink (C4E). F-statistics and p-values for the one-way ANOVAs used to analyze between condition differences are shown within the boxes in each panel. Where the ANOVA was significant, p-values are shown for the between condition Tukey-corrected post-hoc tests. Data are expressed as mean ± SE.

**Figure 4.**
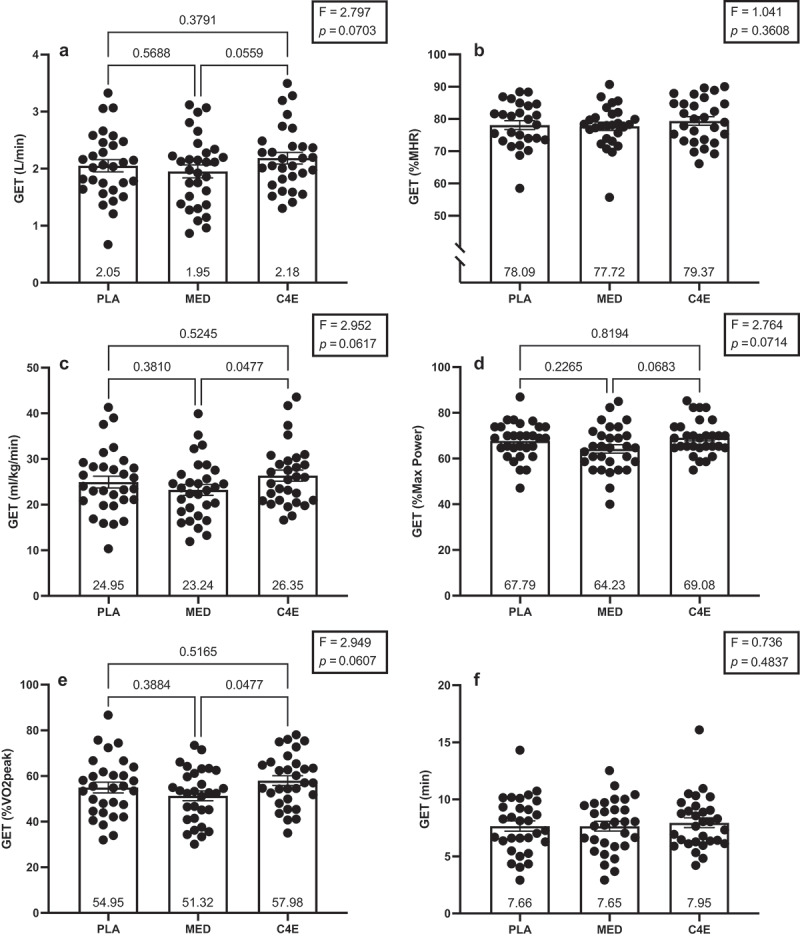
Effects of energy drink consumption on the gas exchange threshold. The gas exchange threshold (GET) expressed in (a) L/min, (b) percent of max heart rate (%MHR), (c) ml/kg/min, (d) percent of max power output (%Max power), (e) percent of $\dot{V}$O_2peak_ (%$\dot{V}$O_2peak_), and (f) time in minutes during the graded exercise test (GXT) following consumption of 16 oz placebo (PLA), monster energy drink (MED), and C4 energy drink (C4E). F-statistics and p-values for the one-way ANOVAs used to analyze between condition differences are shown within the boxes in each panel. Where the ANOVA p-value was ≤ 0.10, p-values are shown for the between condition Tukey-corrected post-hoc tests. Data are expressed as mean ± SE.

There were no significant differences among conditions for $\dot{V}$O_2peak_ (*p* = 0.21), GXT_TTE_ (*p* = 0.21), peak HR (*p* = 0.36), or peak power (*p* = 0.83; Figure 3). However, RER was significantly different among conditions (F_(2, 58)_ = 4.25, *p* = 0.019). Specifically, RER was greater following MED than PLA (+0.030 ± 0.01; *p* = 0.026), whereas no significant differences were observed between MED and C4E (+0.027 ± 0.01; *p* = 0.05) or between C4E and PLA (+0.004 ± 0.01; *p* = 0.94; Figure 3e). MFO was also significantly different among conditions (F_(1.499, 43.48)_ = 6.02, *p* = 0.009, Figure 3f). The MFO was significantly lower following MED compared to both PLA (−0.108 ± 0.02 g/min; *p* < 0.001) and C4E (−0.099 ± 0.04 g/min; *p* = 0.020). There were no differences among conditions for GET when expressed relative to maximal heart rate (%MHR; *p* = 0.36) or as the absolute time (min; *p* = 0.48), and while GET tended to be different among conditions when expressed as absolute (*p* = 0.07) and relative $\dot{V}$O_2_ (*p* = 0.06), when expressed relative to $\dot{V}$O_2peak_ (*p* = 0.06), and when expressed relative to the maximal power output (*p* = 0.07) during the GXT, these differences were also not significant (Figure 4).

### 3.5. ISO_FTG_ test

**Figure 5.**
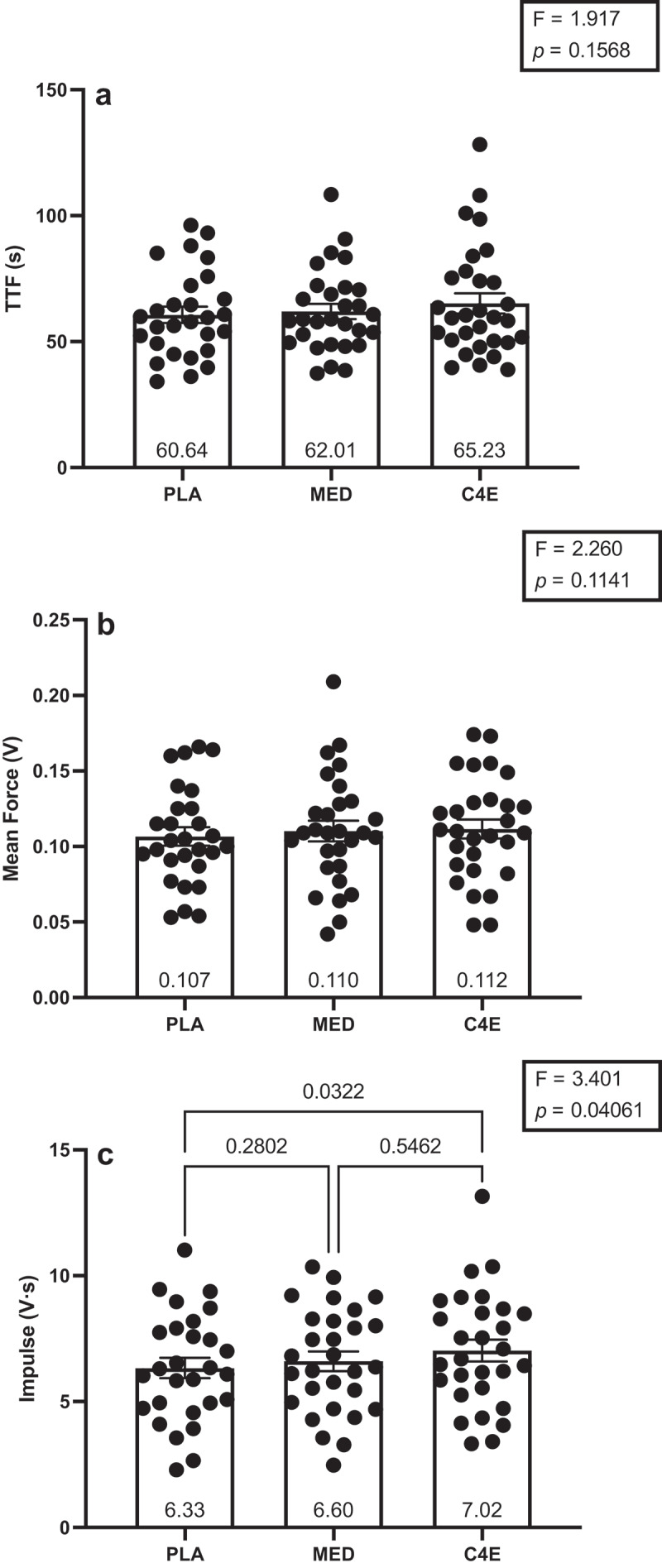
Submaximal isometric fatigue test performance. The (a) time to fatigue (TTF), (b) mean force, and (c) impulse during the isometric fatigue (ISO_FTG_) test following consumption of 16 oz placebo (PLA), monster energy drink (MED), and C4 energy drink (C4E). F-statistics and *p*-values for the one-way ANOVAs used to analyze between condition differences are shown within the boxes in each panel. Where the ANOVA was significant, *p*-values are shown for the between condition Tukey-corrected post-hoc tests. Data are expressed as mean ± SE.

There were no significant differences in TTF_ISO_ and mean force among conditions for either outcome (*p* = 0.16 and 0.11, respectively; Figures 5a,b). However, impulse was significantly different among conditions (F_(2, 54)_ = 3.4, *p* = 0.041, Figure 5c). Impulse was greater for C4E than PLA (+0.658 ± 0.25 V·s; *p* = 0.032).

The MVF condition × time interaction did not reach significance (*p* = 0.054) and forced post-hoc tests revealed no significant differences among conditions (all *p* ≥ 0.08). There was also no condition main effect (*p* = 0.34), but there was a significant main effect for time (F_(3, 87)_ = 23.5, *p* < 0.0001). MVF decreased from baseline to post GXT (−51.6 ± 4.4 N; *p* < 0.0001), increased from post GXT to pre ISO_FTG_ (+33.0 ± 4.4 N; *p* < 0.0001) but was still depressed relative to baseline (−18.7 ± 4.4 N; *p* = 0.0001), and then decreased again from pre ISO_FTG_ to post ISO_FTG_ (−18.0 ± 4.4 N; *p* < 0.0001) such that it was depressed relative to baseline (−36.6 ± 4.4 N; *p* < 0.0001).

**Figure 6.**
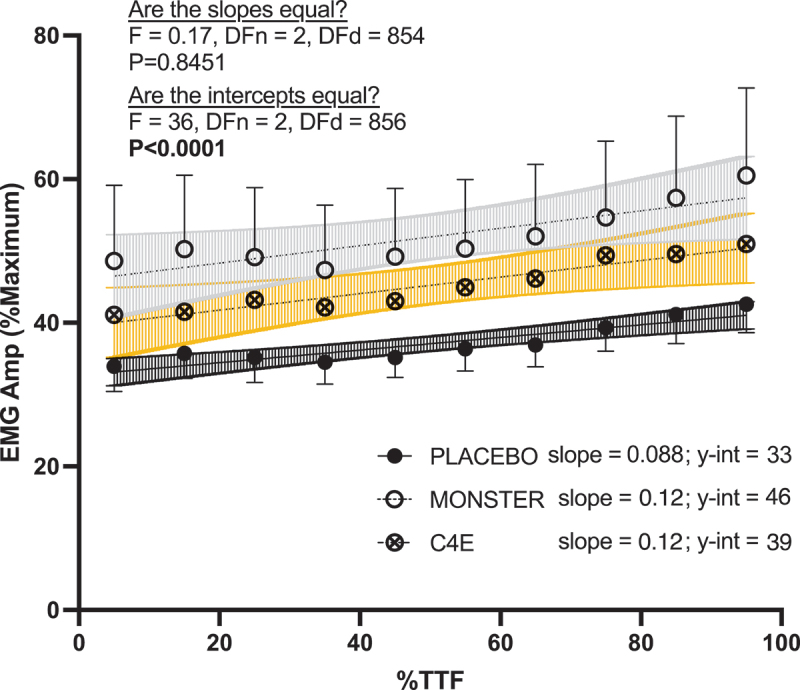
Effects of energy drink consumption on the EMG amplitude versus time. F-statistics and p-values of the tests for between condition differences in slopes and y-intercepts are shown.

Finally, the condition × time interaction for EMG_AMP_ during the ISO_FTG_ test was not significant (*p* = 0.22), but there were significant main effects for time (F_(1.908, 55.34)_ = 19.22, *p* < 0.0001) and condition (F_(1.923, 55.75)_ = 5.65, *p* = 0.006). Follow-up linear regression analyses confirmed that the slopes of the EMG_AMP_ versus normalized time relationships were similar among conditions, indicating that EMG_AMP_ increased similarly across time for all conditions. However, the y-intercepts of the EMG_AMP_ versus normalized time relationships were different and indicated that EMG_AMP_ was greater in the C4E (39 ± 5%) and MED (46 ± 6%) conditions than in PLA (33 ± 2%) independent of time (Figure 6).

### 3.6. Leg blood flow

No significant condition × time interaction was observed for LBF (*p* = 0.37), artery cross-sectional area (*p* = 0.85), or mean blood flow velocity (*p* = 0.27). There were also no significant main effects for condition for any of these measures (*p* = 0.98, 0.36, and 0.84, respectively). However, there were main effects for time for LBF (F_(1.474, 42.75)_ = 93.28, *p* < 0.0001), artery cross-sectional area (F_(1.837, 52.28)_ = 13.09, *p* < 0.0001), and mean blood flow velocity (F_(1.443, 41.84)_ = 75.74, *p* < 0.0001). The means ± SD for each timepoint independent of condition are presented in Table 4.

**Table 4.** Profile of mood states and session RPE following beverage consumption.

	PLA	MED	C4E	*p*-value
Total Mood Disturbance	−.03 ± 1.33	0.57 ± 1.21	0.17 ± 1.52	0.94
Anger-Hostility	0.27 ± .13	0.23 ± 0.09	0.23 ± 0.09	0.96
Confusion-Bewilderment	0.63 ± .21	0.70 ± 0.20	0.87 ± 0.27	0.63
Depression-Dejection	0.10 ± .07	0.40 ± 0.17	0.37 ± 0.21	0.22
Fatigue-Inertia	7.07 ± .62	6.83 ± 0.62	7.17 ± 0.73	0.86
Tension-Anxiety	1.20 ± .30	1.10 ± 0.30	1.07 ± 0.31	0.88
Vigor-Activity	8.90 ± .72	8.70 ± 0.74	9.53 ± 0.76	0.21
Friendliness	9.90 ± .76	10.33 ± 0.89	10.50 ± 0.92	0.40
Session RPE	7.7 ± 1.9	7.0 ± 2.4	7.4 ± 2.1	0.09

All data are presented as mean ± SE. RPE = rating of perceived exertion; PLA = Placebo; MED = Monster Energy Caffeine Drink; C4E = C4 Energy Drink.

### 3.7. Blood pressure and heart rate

**Figure 7.**
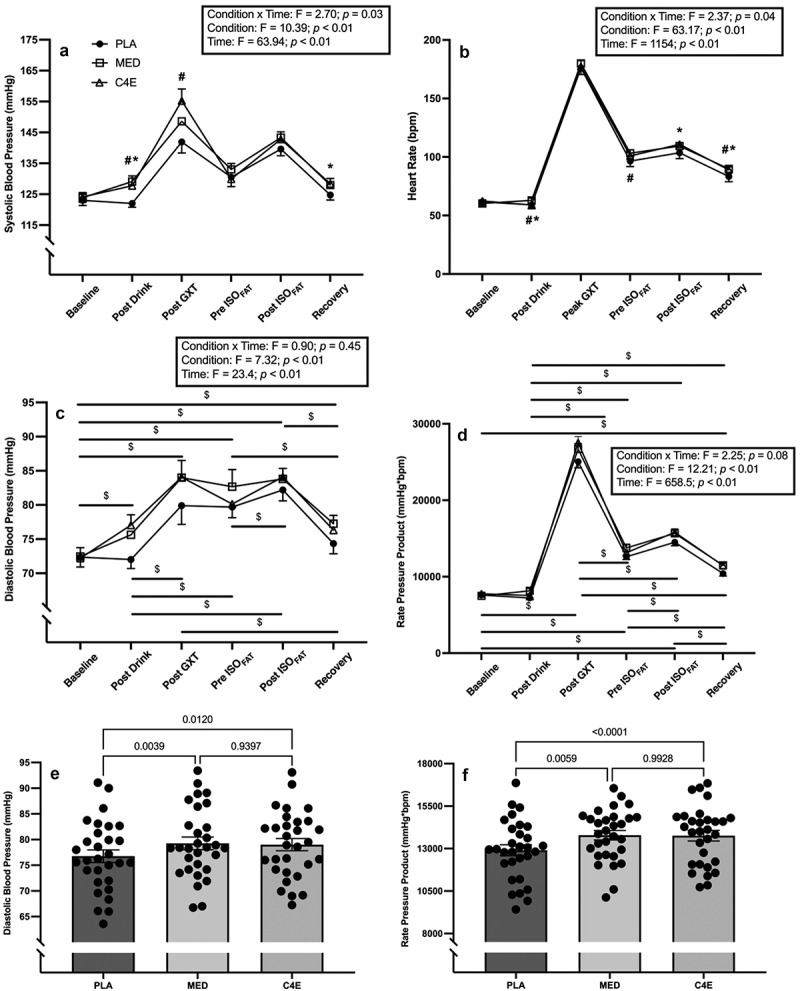
Cardiovascular hemodynamic responses to energy drink consumption. (a) Systolic blood pressure, (b) heart rate, (c) diastolic blood pressure, (d) rate pressure product, (e) diastolic blood pressure condition main effect, and (f) rate pressure product condition main effect readings were taken 6 times at each experimental visit. At each visit, participants consumed 16 oz of either placebo (PLA), monster energy drink (MED), or C4 energy (C4E). Heart rate and blood pressure was taken before drink consumption (baseline), 40 minutes after drink consumption (post drink), immediately before and immediately after an isometric fatigue test (pre ISO_FTG_ and post ISO_FTG_, respectively), and 10 minutes after an isometric fatigue test (recovery). Peak heart rate values observed during a maximal graded exercise test are also presented (peak GXT), while blood pressure was taken immediately after a GXT (post GXT). * = significant difference between PLA and MED; # = significant difference between PLA and C4E; $ = significant difference between timepoints collapsed across condition. Data are expressed as mean ± SE.

There was a significant condition × time interaction for HR (F_(5.376, 154.3)_ = 2.4, *p* = 0.037). There was no difference in HR at baseline across conditions, but post-drink HR was significantly higher in the MED versus both the PLA (+3.9 ± 1.5 bpm; *p* = 0.035) and the C4E (+4.0 ± 1.2 bpm; *p* = 0.010) conditions. Pre-ISO_FTG_ HR was significantly higher in the MED versus the PLA condition (+6.7 ± 2.6 bpm; *p* = 0.034) but was not different in the C4E versus PLA condition (+4.4 ± 2.0 bpm; *p* = 0.09). Post-ISO_FTG_ HR was significantly higher in the C4E versus the PLA condition (+7.3 ± 2.2 bpm; *p* = 0.007), whereas the difference in HR between MED and PLA was not significant (+5.8 ± 2.4 bpm; *p* = 0.05). Finally, at recovery, HR was significantly higher in the MED (+6.5 ± 1.5 bpm; *p* < 0.001) and C4E (+5.7 ± 1.9 bpm; *p* = 0.015) conditions relative to PLA. There was also a significant condition × time interaction for SBP (F_(4.095, 117.5)_ = 2.7, *p* = 0.033). Post hoc tests revealed that SBP did not differ at baseline, pre-ISO_FTG_, or post-ISO_FTG_ among conditions. However, SBP was significantly greater in the MED (+7.1 ± 1.2 mmHg; *p* < 0.001) and C4E (+5.7 ± 1.0 mmHg; *p* < 0.001) versus the PLA conditions post-drink consumption. Post-GXT, SBP was greater in the C4E versus the PLA condition (+13.3 ± 3.8 mmHg; *p* < 0.001), but there was no difference in SBP between MED and PLA (*p* = 0.35). During recovery, SBP was significantly greater in the MED versus PLA condition (+3.2 ± 1.1 mmHg; *p* = 0.014), and there was no difference between C4E and PLA (*p* = 0.08). No significant condition × time interaction was observed for DBP (*p* = 0.45). However, there were main effects for condition (F_(1.982, 57.46)_ = 7.3, *p* = 0.002) and time (F_(2.06, 59.83)_ = 23.4, *p* < 0.0001). Independent of time, DBP was significantly higher in the MED (+2.50 ± 0.71 mmHg; *p* = 0.004) and C4E (+2.24 ± 0.73 mmHg; *p* = 0.012) versus the PLA conditions. Independent of condition, DBP increased from baseline to post-drink, from post-drink to post-GXT, and then remained elevated through post-ISO_FTG_ before decreasing in recovery whilst remaining elevated relative to baseline. All post-hoc comparisons can be seen in Figure 7c.

No significant condition × time interaction was observed for RPP (*p* = 0.08). However, there were main effects for condition (F_(1.631, 47.29)_ = 12.21, *p* = 0.0001) and time (F_(2.353, 68.24)_ = 658.5, *p* < 0.0001). Independent of time, RPP was significantly higher in the MED (+882.9 ± 262.1 mmHg*bpm; *p=*0.006) and C4E (+857.0 ± 159.6 mmHg*bpm; *p* < 0.0001) versus the PLA conditions Figure 7f. Independent of condition, RPP did not change from baseline to post-drink but then increased post-GXT, decreased to pre-ISO_FTG_ while remaining elevated relative to baseline, increased to post-ISO_FTG_, and then decreased again in recovery while remaining elevated relative to baseline. All post-hoc comparisons are depicted in Figure 7d.

### 3.8. Electrocardiogram

**Figure 8.**
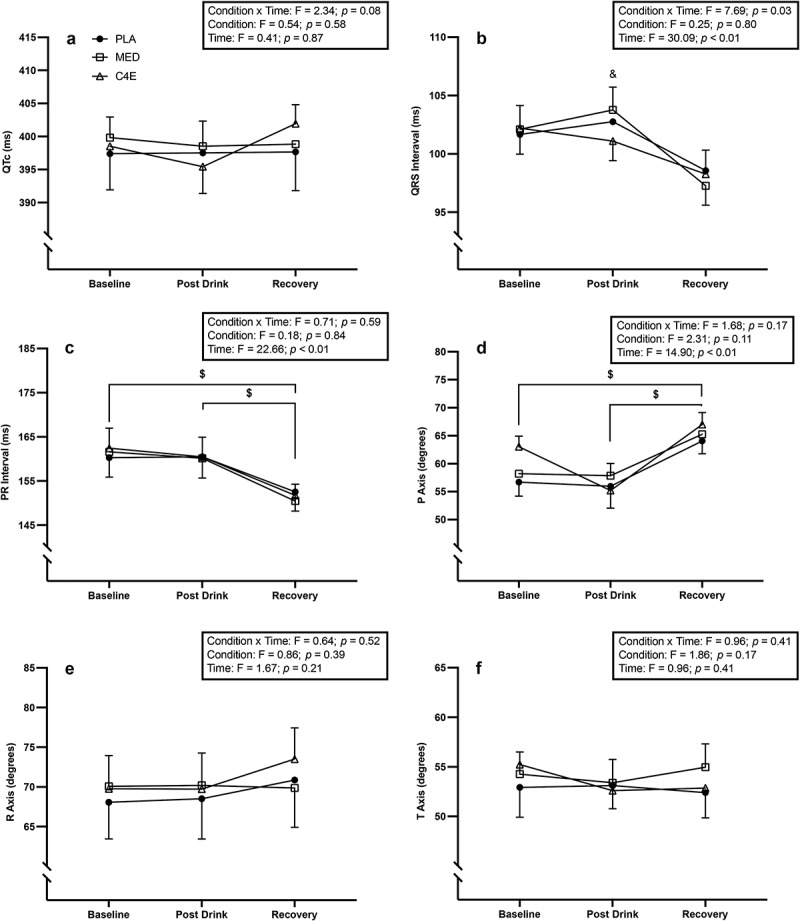
Effects of energy drink consumption on electrocardiogram (ECG) characteristics. The (a) corrected QT interval (QTc) (b) QRS interval, (c) PR interval, (d) P axis, (e) R axis, and (f) T axis are displayed above. F-statistics and p-values for the one-way ANOVAs used to analyze between condition differences are shown within the boxes in each panel. Where the ANOVA was significant, p-values are shown for the between condition Tukey-corrected post-hoc tests. & = significant difference between MED and C4 energy (C4E); $ = significant difference between timepoints collapsed across condition. Data are expressed as mean ± SE.

There were no significant condition × time interactions, nor condition or time main effects for QTc, R, or T axis (all *p* ≥0.08) (Figure 8). There was a significant condition × time interaction for QRS duration (F_(4, 115)_ = 2.79, *p* = 0.03; Figure 8b). Post hoc tests revealed that QRS duration was significantly longer in MED (+2.67 ± 1.06 ms; *p* = 0.03) post-drink consumption compared to C4E, but was not significantly different in any other comparison. There was no condition × time interaction for PR interval duration (*p* = 0.59) or P axis (*p* = 0.17), but there was a main effect for time for both (F_(2, 58)_ = 22.66, *p* < 0.001 and F_(2, 58)_ = 14.9, *p* < 0.001; Figure 8c,d, respectively). PR interval duration was shorter at recovery compared to both baseline (−9.87 ± 1.28 ms; *p* < 0.001 and post-drink consumption (−8.78 ± 1.38 ms; *p* < 0.001). The P axis was greater in recovery than at baseline (6.13 ± 1.35°; *p* < 0.001) or post-drink (9.1 ± 1.55°; *p* < 0.001).

### 3.9. Mood

There were no differences in Total Mood Disturbance, Fatigue-Inertia, Vigor-Activity, Tension-Anxiety, Depression-Dejection, Anger-Hostility, or Confusion-Bewilderment scores observed among conditions (all *p* ≥0.21). There was also no difference in session RPE observed among conditions (*p* = 0.09). Data are presented in Table 4.

## 4. Discussion

We investigated the acute effects of a novel, non-caloric caffeinated energy drink (C4E) relative to a traditional, high-sugar caffeinated energy drink (MED) and PLA control on exercise performance, leg blood flow, and cardiovascular safety. In the present study, we demonstrated that acute MED consumption increased exercising RER and decreased MFO relative to PLA, but did not influence exercise performance. In contrast, C4E did not alter RER or MFO relative to PLA. Further, C4E improved impulse during a sub-maximal isometric fatigue test by 9.9% relative to PLA. Neither MED nor C4E influenced resting or post-exercise leg blood flow. Finally, both C4E and MED caused resting SBP, DBP, and HR to increase, while C4E elicited an increased SBP immediately following exercise compared to PLA but not MED. Except for an increase in QRS duration in MED compared to C4E, there were no effects of acute C4E or MED consumption on QTc interval length, nor on any other ECG characteristics, mood, or perceived effort during the testing session. Together, these results suggest that C4E may improve semi-fasted resistance exercise performance in prolonged, exhaustive exercise bouts without disrupting fuel utilization.

### 4.1. Exercise performance and blood flow

Our results indicate that neither C4E nor MED influence maximal exercise outcomes during a GXT to volitional exhaustion, including peak heart rate, peak power, TTE, and $\dot{V}$O_2peak_. Few studies have examined the effects of low-calorie, caffeine-containing energy drinks on GXT performance [13,42]. Similarly, few studies have examined the effects of caloric, caffeinated energy drinks on GXT performance and, similar to the present study, Al-Fares *et al*. reported no effects on peak HR, TTE or, $\dot{V}$O_2peak_ [43]. Interestingly, several prior studies have instead indicated that caffeinated energy drinks are efficacious for improving endurance exercise performance when performed at sub-maximal work rates or during time-trials [3–5]. Thus, together, our data in combination with these previous studies suggest that, in young, adult men under semi-fasted conditions, neither caloric, nor non-caloric caffeinated energy drinks differentially affect maximal endurance exercise performance during graded exercise tests performed to volitional exhaustion. However, additional studies are needed to understand the effects of caloric versus non-caloric energy drink consumption on time trial and sub-maximal endurance exercise performance. It would also be important to understand how energy drink consumption would influence exercise performance in the face of various dietary or behavioral stressors, such as following prolonged fasting or after sleep restriction/deficiency.

Despite observing that C4E and MED had no effects on maximal endurance performance parameters, MED consumption reduced MFO during the GXT compared to both C4E and PLA, which was accompanied with an average RER that was greater than PLA (+3.1%, *p* = 0.03) but not C4E (+2.7%, *p* = 0.057). At submaximal exercise intensities, energy demands are primarily met via the oxidation of lipids with a lesser contribution of carbohydrates. However, as the exercise intensity increases, the contribution from lipids decreases while the contributions from carbohydrate increase [44]. There are few data available regarding the impact of acute energy drink consumption and fuel utilization during exercise, but carbohydrate consumption prior to exercise has a suppressive effect on fat oxidation and MFO. In support, Achten *et al*. reported a 28% decrease (0.46 vs 0.33 g/min) in MFO during a maximal GXT 45 min after the consuming 75 g of carbohydrates versus placebo [29]. Nelson *et al*. provided a MED at the volume necessary to provide 2 mg/kg of caffeine to 15 healthy recreationally active male and female participants 30-min prior to a maximal exercise test at 100% ventilatory threshold, and also reported higher RERs compared to placebo [45]. Nienhueser *et al*. provided three different commercial energy drinks containing 27–31 g of sugar to 10 healthy male participants 45 min before a 15-min treadmill run at 50% $\dot{V}O2$ and reported a 10–15% increase in RER compared to placebo [46]. In the current study, unlike PLA and C4E, MED contained 58 g of carbohydrates, which likely impacted fuel utilization by promoting a greater contribution of carbohydrates to meet energy demands [47], thus driving RER up and attenuating MFO. Interestingly, however, the increased availability of carbohydrates from MED did not positively impact markers of exercise performance under semi-fasted conditions.

There is limited prior work examining the efficacy of caffeinated energy drink consumption on the GET. Interestingly, GET (expressed relative to absolute and relative peak oxygen consumption) was 12–13% greater (*p* = 0.047–0.06) following C4E than MED consumption, but not different from PLA in the present study. It should be noted, however, that these differences were observed in forced post-hoc tests as the main effects for condition did not reach significance (*p* = 0.06–0.07). While there is a paucity of data regarding the acute effects of energy drinks on anaerobic thresholds, caffeine consumption alone reportedly does not have a strong influence [9,48,49]. There are also little data available regarding the effects of carbohydrate consumption on GET using pulmonary gas analyses as in this study, but there are data regarding the effects on lactate threshold. A 3-day high-carbohydrate diet has been shown to reduce the $\dot{V}O2$ at the onset of blood lactate accumulation [50]. It has also been shown that when free fatty acid concentrations are elevated, lactate threshold is increased [51]. Yet, acute pre-exercise carbohydrate consumption has been shown to have no effect on lactate threshold [52,53]. Therefore, our data generally agree and extend the current body of literature to suggest that acute energy drink consumption does not strongly influence the GET, and any potential differences in GET in this study are likely due to the aforementioned differences in substrate availability and utilization, and subsequently greater CO_2_ production following MED consumption. This is further supported by our data indicating that neither C4E nor MED augmented leg blood flow, and thus were not likely to have influenced blood supply and oxygen delivery [54]. Given the mechanism of action for several of the active ingredients in C4E that may require chronic supplementation, it would be beneficial for future studies to examine the efficacy of sub-chronic or chronic C4E consumption on GET.

In the present study, C4E improved impulse – a surrogate of total work capacity during isometric muscle actions – relative to PLA during the fatiguing submaximal, isometric leg extension exercise bout completed after the GXT. In contrast, MED had no effect on impulse. These effects on impulse were realized despite C4E having no significant impacts on either MVF or TTF. In other words, the subtle improvements in MVF and TTF associated with C4E consumption were not statistically significant, but were substantial enough to improve total impulse, which is the product of MVF and TTF. Prior studies examining the efficacy of energy drink consumption on muscular strength and endurance have been inconsistent, with some reporting energy drink-related improvements [6–8], and others reporting no effects [11,12,55]. Chtourou *et al*. reported a significantly greater handgrip force (58.2 vs. 55.5 kg) following consumption of Red Bull in 19 physically active young men [56]. Similarly, Astley *et al*. [8] reported an increase in handgrip strength following energy drink consumption (53.7 vs. 47.7 kg). While no studies have examined isometric leg extension fatiguability following energy drink consumption, Astley *et al*. [8] reported that energy drink consumption promoted the completion of more repetitions to failure (11.5 vs. 9.5 reps) during leg extension exercise at 80% of one repetition maximum. Unlike the present study, the aforementioned studies utilized energy drinks that contained moderate calories and substantial added sugars, whereas the present study examined the effects of a sugar-free energy drink on isometric leg extension performance. Notably, our data show that while C4E promoted improved leg extension fatiguability, MED did not. Thus, acute C4E consumption may improve force-generating capacity and total impulse (work) in resistance-type tasks in the midst of exhaustive exercise bouts.

### 4.2. Cardiovascular safety

The current study showed that C4E and MED both increased SBP, while MED increased QRS interval length compared to C4E. Furthermore, neither drink altered QTc, PR interval, P axis, R axis, or T axis compared to PLA. While DBP and RPP were greater in the C4E and MED conditions than during PLA, these effects were independent of time. Whereas this may suggest that the increased DBP and RPP in C4E and MED were not caused by C4E and MED consumption per se, it is worth noting that there were no differences in DBP or RPP among conditions (*p* = 0.96 and 0.39) at baseline. Therefore, combined with the effect on SBP, our interpretation is that this condition main effect was driven by the effects of C4E and MED consumption. In a 2016 meta-analysis, Shah *et al*. [57] reported that energy drinks containing ≥200 mg of caffeine promote 6.4 mmHg (95% CI = 4.6–8.3) increases in resting SBP, while those containing <200 mg caffeine promote 3.7 (95% CI = 1.7–5.8) mmHg increases. In the present study, C4E (200 mg caffeine) produced a resting increase in SBP of 5.7 mmHg, whereas MED (160 mg caffeine) produced a resting increase of 7.1 mmHg. Consequently, the increase in resting SBP following C4E consumption was as expected given the dose of caffeine provided, whereas the increase in resting SBP following MED was greater than expected. Prior studies have demonstrated that the combination of ingredients in traditional energy drinks (e.g. caffeine, taurine, glucuronolactone) have cardiovascular effects that are different from the ingredients in isolation [21]. Thus, it is plausible that the greater than expected effect of MED on resting SBP was due to the combined effects of the active ingredients in MED. Still, our study showed no influence of MED or C4E consumption on the QTc interval, suggesting that acute consumption of a standard, 16 oz energy drink with ≤200 mg of caffeine produces no adverse effects on ventricular repolarization.

Uniquely, our study design also provided the opportunity to examine the influence of C4E and MED consumption on hemodynamic and ECG parameters following exhaustive exercise and during passive recovery. C4E consumption increased SBP following the GXT, and increased HR after the isometric exercise bout and in recovery. MED consumption increased SBP in recovery and produced increases in HR before the start of isometric exercise and in recovery. Thus, both C4E and MED generally promoted increases in BP and HR following exercise that persisted into recovery. These findings are consistent with prior studies showing that caffeine consumption alone can disrupt post-exercise autonomic recovery because of increased sympathetic activity [25].

### 4.3. Limitations

There are several limitations to our study. First, the study population included men alone and as such the results are not generalizable to women. The decision to proceed with men alone was based primarily on existing evidence that caffeine metabolism is affected by menstrual cycle phase {1483492}. Whereas the repeated-measures crossover design was a notable strength, it also introduced logistical difficulties whereby controlling for menstrual cycle phase would have required prolonged washout periods between treatments. In addition, the relative dosing of study ingredients would have been different, on average, for men versus women. Future studies will be necessary to examine the metabolic, cardiovascular, and neuromuscular effects of novel energy drink formulations in women. These studies may wish to similarly use cross-over designs but test irrespective of menstrual cycle phase, with the assumption that randomization will necessarily control for this effect. However, it is also possible that this will increase variability between repeated measures and thus may require larger sample sizes than those used herein. In addition, if sex-differences are of interest, it will be necessary to match ingredient dosing relative to bodyweight or attempt to match males and females for bodyweight and body composition. As previously mentioned, several of the primary ingredients in C4S exert their greatest effects following short- (e.g. ≥1 week for L-citrulline) to longer-term supplementation (e.g. ≥4 weeks for beta-alanine). Thus, future studies may wish to examine the effects of habitual C4S consumption on exercise performance. Further, whereas the acute effects of high-dose energy drink consumption on cardiovascular safety have been described, the effects of chronic consumption on cardiovascular and cardiometabolic health are unclear. Future studies may choose to examine the effects of chronic novel, non-caloric versus caloric energy drink consumption on cardiovascular and cardiometabolic health.

## 5. Conclusions

In conclusion, our data indicate that acute MED consumption increases RER and attenuates MFO and has no influence on the anaerobic threshold, $\dot{V}$O_2peak_, or performance during a fatiguing isometric work bout. In contrast, C4E consumption did not disrupt semi-fasted RER or MFO but enhanced impulse relative to PLA during the fatiguing isometric work bout. Neither MED nor C4E influenced mood or leg blood flow. Furthermore, both MED and C4E elicited increases in resting SBP and heart rate, likely increased DBP and RPP, but had no effect on ventricular repolarization or on other indicators of cardiac electrical activity. Consequently, our data suggest that acute C4E consumption enhances force-generating capacity in the context of semi-fasted, exhaustive exercise bouts without disrupting fuel utilization. Finally, energy drinks such as C4E contain multiple ingredients that likely improve exercise performance when consumed habitually (e.g. beta-alanine and L-citrulline). However, it is still unclear how habitual consumption of sugar-free or traditional energy drinks such as C4E or MED may, respectively, influence cardiovascular or metabolic health. Therefore, future studies should examine the efficacy of chronic energy drink consumption on exercise performance while considering cardiovascular and metabolic health outcomes.

## Supplementary Material

Supplemental Material
