# Supplementary material for: Acute effects of commercial energy drink consumption on exercise performance and cardiovascular safety: a randomized, double-blind, placebo-controlled, crossover trial
Source: J Int Soc Sports Nutr. 2024 Jan 10;21(1):2297988. doi: 10.1080/15502783.2023.2297988 (PMC10783828; doi:10.1080/15502783.2023.2297988)
Supplement: Supplemental Material [file RSSN_A_2297988_SM5291.docx]

| **Supplemental Table 1.** Nutrition Facts panels with Ingredients List obtained from the labels of the non-caloric and sugar-free Energy Drink (C4E; C4 Energy®, Nutrabolt®, Austin, TX) and the high calorie, high-sugar Energy Drink (MED; Monster Energy®, Monster Energy Company, Corona, CA). | | |
| --- | --- | --- |
|  | MED | C4E |
| Can Size (fl. oz.) | 16 | 16 |
| Kcal | 230 | 0 |
| Fat (g) | 0 | 0 |
| Carbohydrates (g) | 58 | 0 |
| Sugars (g) | 54 | 0 |
| Sodium (mg) | 370 | 0 |
| Protein (g) | 0 | 0 |
| Niacin (% of daily value) | 200 | 190 |
| Vitamin B12 (% of daily value) | 200 | 250 |
| Riboflavin (% of daily value) | 200 | 0 |
| Vitamin B6 (% of daily value) | 200 | 0 |
| **Monster Energy Caffeine Drink (MED) Ingredients:** Carbonated Water, Sugar, Glucose, Citric Acid, Natural Flavors, Taurine, Sodium Citrate, Panax Ginseng Flavor, L-Carnitine L-Tartrate, Caffeine, Sorbic Acid (preservative), Color Added, Niacinamide (Vit. B3), Sucralose, Salt, Inositol, Pyridoxine Hydrochloride (Vit. B6), Riboflavin (Vit. B2), Maltodextrin, Cyanocobalamin (Vit. B12).  **C4 Energy (C4E) Ingredients:** Carbonated Water, Carnosyn®, Beta-Alanine, L-Citrulline, Malic Acid, BetaPower®, Beta Anhydrous, Citric Acid, Natural Flavors, Tartaric Acid, Potassium Sorbate (preservative), Caffeine Anhydrous, Sucralose, Acesulfame Potassium, N-Acetyl-L-Tyrosine, Niacinamide, Cyanocobalamin (Vitamin B12). | | |
